# Exposure to bilingual or monolingual maternal speech during pregnancy affects the neurophysiological encoding of speech sounds in neonates differently

**DOI:** 10.3389/fnhum.2024.1379660

**Published:** 2024-05-22

**Authors:** Natàlia Gorina-Careta, Sonia Arenillas-Alcón, Marta Puertollano, Alejandro Mondéjar-Segovia, Siham Ijjou-Kadiri, Jordi Costa-Faidella, María Dolores Gómez-Roig, Carles Escera

**Affiliations:** ^1^Brainlab – Cognitive Neuroscience Research Group, Departament de Psicologia Clinica i Psicobiologia, Universitat de Barcelona, Barcelona, Spain; ^2^Institut de Neurociènces, Universitat de Barcelona, Barcelona, Spain; ^3^Institut de Recerca Sant Joan de Déu, Esplugues de Llobregat, Barcelona, Spain; ^4^BCNatal – Barcelona Center for Maternal Fetal and Neonatal Medicine (Hospital Sant Joan de Déu and Hospital Clínic), University of Barcelona, Barcelona, Spain

**Keywords:** speech brainstem responses, bilingualism, newborns, early language acquisition, frequency-following response (FFR), prenatal exposure

## Abstract

**Introduction:**

Exposure to maternal speech during the prenatal period shapes speech perception and linguistic preferences, allowing neonates to recognize stories heard frequently *in utero* and demonstrating an enhanced preference for their mother’s voice and native language. Yet, with a high prevalence of bilingualism worldwide, it remains an open question whether monolingual or bilingual maternal speech during pregnancy influence differently the fetus’ neural mechanisms underlying speech sound encoding.

**Methods:**

In the present study, the frequency-following response (FFR), an auditory evoked potential that reflects the complex spectrotemporal dynamics of speech sounds, was recorded to a two-vowel /oa/ stimulus in a sample of 129 healthy term neonates within 1 to 3 days after birth. Newborns were divided into two groups according to maternal language usage during the last trimester of gestation (monolingual; bilingual). Spectral amplitudes and spectral signal-to-noise ratios (SNR) at the stimulus fundamental (F_0_) and first formant (F_1_) frequencies of each vowel were, respectively, taken as measures of pitch and formant structure neural encoding.

**Results:**

Our results reveal that while spectral amplitudes at F0 did not differ between groups, neonates from bilingual mothers exhibited a lower spectral SNR. Additionally, monolingually exposed neonates exhibited a higher spectral amplitude and SNR at F_1_ frequencies.

**Discussion:**

We interpret our results under the consideration that bilingual maternal speech, as compared to monolingual, is characterized by a greater complexity in the speech sound signal, rendering newborns from bilingual mothers more sensitive to a wider range of speech frequencies without generating a particularly strong response at any of them. Our results contribute to an expanding body of research indicating the influence of prenatal experiences on language acquisition and underscore the necessity of including prenatal language exposure in developmental studies on language acquisition, a variable often overlooked yet capable of influencing research outcomes.

## Introduction

The process of language acquisition has long been a point of uncertainty in research exploring the roots of human language. Researchers have conducted extensive investigations to understand the initial state and process of language acquisition, providing insights into how environmental and genetic factors interact to fashion language and cognitive function, and the mechanisms underlying brain plasticity (Weaver et al., 2004; Werker and Tees, 2005; Barkat et al., 2011; Werker and Hensch, 2015). It is now widely accepted that both genetic and experiential factors contribute to language acquisition (Werker and Curtin, 2005; Gervain and Mehler, 2010), and researchers are interested in understanding how these factors interact during human development.

Infants at birth already exhibit advanced speech perception and language learning abilities. Newborns manifest a preference for speech over non-speech sounds (Vouloumanos and Werker, 2007), can discriminate between different languages based on their speech rhythms (Ramus et al., 2000), detect word boundaries (Christophe et al., 2001), discriminate words with different patterns of stress (Sansavini et al., 1997), or even distinguish consonant sounds (Cabrera and Gervain, 2020) and encode voice pitch in an adult-like manner (Arenillas-Alcón et al., 2021). These findings support the role of a genetically driven cerebral organization towards processing specific speech characteristics.

However, the prenatal period is not devoid of language experience and the study of its influence on the newborn’s speech and language encoding capacities is receiving increasing attention. Hearing becomes functional and undergoes most of its development around the 26th to 28th week of gestation, allowing the fetus to perceive the maternal speech signal (Ruben, 1995; Moore and Linthicum, 2007; Granier-Deferre et al., 2011; May et al., 2011; Anbuhl et al., 2016). Although the exact characteristics of the acoustic signal reaching the fetus are not fully understood, intrauterine recordings from animal models and simulations suggest that the maternal womb acts as a low-pass filter, attenuating around 30 dB for frequencies over 600–1,000 Hz (Gerhardt and Abrams, 2000). The low-frequency components of speech that are transmitted through the uterus include pitch, slow aspects of rhythm and some phonetic information (Moon and Fifer, 2000; May et al., 2011). Evidence indicates that prenatal exposure to speech, despite attenuated by the filtering properties of the womb, shapes speech perception and linguistic preferences of newborns, as shown by studies revealing that neonates can recognize a story heard frequently *in utero* (DeCasper and Spence, 1986), prefer the voice of their mother (DeCasper and Fifer, 1980) and prefer their native language (Moon et al., 1993). Additionally, prenatal learning extends beyond these common preferences. Recent findings indicate that infants acquire specific knowledge of the prosody (Gervain, 2018) and prefer the rhythmic patterns of the language they were exposed to while *in utero* (Mariani et al., 2023), indicating a very early specialization for their native language.

Yet, with reported rates of bilingualism of around 65% in Europe (Luk, 2017), an open question remains on the influence of prenatal exposure to more than one language on neural plasticity. Over the past 20 years, mounting evidence has suggested that both exposure to a bilingual acoustic environment and learning several languages affects not only language acquisition but a wide range of developmental processes including perception, cognition and brain development (Byers-Heinlein et al., 2019). Prior research has highlighted that early exposure to language influences infants’ acquisition of speech sounds, indicating that, at birth, infants are able to discriminate all phonetic contrasts. As infants age, their perceptual systems are tuned to collapse over phonetic contrasts not found in the input language or languages, such that their ability to distinguish between phonetic elements becomes increasingly specific to their native language(s) (Kuhl et al., 2006; Saffran et al., 2006; Gervain and Werker, 2008; Kovács and Mehler, 2009; Bosch and Sebastián-Gallés, 2010). Moreover, cross-language interactions modulate almost every level of language processing, including speech perception, phonological, vocabulary and semantic development [for comprehensive review, refer to Hammer et al. (2014) and Kroll et al. (2012)]. Furthermore, some bilinguals switch from one language to the other within the same sentence, demonstrating greater demands on cognitive control than monolinguals to navigate the potential cross-language competition considering that language production is equivalent (Kovács and Mehler, 2009).

Speaking two languages daily also has consequences for the way in which higher cognitive processes operate and results in more precocious development of inhibition and attentional abilities (Costa et al., 2008; Kovács and Mehler, 2009; for review see Barac et al., 2014; Bialystok, 2017). There is evidence for functional and structural brain changes associated with bilingualism, even after brief periods of second-language learning (for extensive review see Li et al., 2014). Bilingual infants show different brain responses to native and non-native speech sounds than monolingual infants (Conboy and Kuhl, 2011). Bilingualism also affects the structure of both grey (Ressel et al., 2012) and white matter (Kuhl et al., 2016) in adults. The observed advantages in cognitive control and attentional abilities, as well as the pattern of structural differences, are modulated by the age of second language acquisition, whether the two languages were acquired simultaneously from birth or sequentially later in life and the interaction between languages (Kroll et al., 2012; Barac et al., 2014; Li et al., 2014).

As bilingual mothers speak using two different sets of phonemic categories and even use two slightly different voice pitch ranges (e.g., Ordin and Mennen, 2017), *in-utero* bilingual environments are characterized by a greater complexity of the reaching speech signal than monolingual ones. Interestingly, neonates exposed prenatally to a bilingual environment can discriminate their two native languages already at birth and exhibit equal preferences for both (Byers-Heinlein et al., 2010). Thus, it appears clear that linguistic experiences while *in utero* play a significant role in shaping the early development of speech processing. However, how different prenatal maternal linguistic exposure influences the neural mechanisms underlying speech sound processing at birth is currently unknown.

A large body of evidence has supported the study of the neural encoding of speech sounds through electrophysiological recordings. In particular, the frequency-following response (FFR) can provide insights into the underlying neural mechanisms associated with prenatal language experience, shedding light on how early linguistic exposure shapes the speech-encoding capacities of newborns. The FFR is an auditory evoked potential elicited by periodic complex sounds that reflects neural synchronization with the auditory eliciting signal along the ascending auditory pathway (Skoe and Kraus, 2010; Krizman and Kraus, 2019), providing an accurate snapshot of the neural encoding of speech sounds. FFR recordings have thus become a useful tool to investigate the ability to distinguish between the pitch of different speakers’ voices and the ability to encode the fine spectrotemporal details that distinguish different voiced speech sounds (Gorina-Careta et al., 2022). The interest in the neonatal FFR arises from its potential to serve as a predictive measure for future language development (Schochat et al., 2017), since alterations in FFR patterns in children have been associated with difficulties in reading and learning, dyslexia, impairments in phonological awareness and even autism (King et al., 2002; Banai et al., 2009; Chandrasekaran et al., 2009; Basu et al., 2010; Hornickel et al., 2012; Lam et al., 2017; Otto-Meyer et al., 2018; Font-Alaminos et al., 2020; Rosenthal, 2020). Interestingly, the FFR reflects the impact of a wide range of auditory experiences in children and adults, including training interventions, musical practice and bilingualism (Russo et al., 2005; Song et al., 2008; Kraus and Chandrasekaran, 2010; Krizman et al., 2012, 2015; Carcagno and Plack, 2017; Skoe et al., 2017; Gorina-Careta et al., 2019). In adults it has been observed that bilingual experience enhances the neural responses to the fundamental frequency of sounds (Krizman et al., 2015; Skoe et al., 2017), as well as the subcortical representation of pitch-relevant information (Krizman et al., 2012) and neural consistency, which correlated with both a better attentional control and language proficiency (Krizman et al., 2014). In neonates, FFR recordings have also been used to study the effects at birth of prenatal fetal auditory experiences such as music exposure (Arenillas-Alcón et al., 2023), but the influence of prenatal maternal bilingual speech remains unexplored.

In the present study, we aimed to examine the influence of maternal bilingual linguistic exposure *in-utero* in speech sound encoding at birth. To that end, we recorded FFRs from newborns who had been exposed to either a monolingual or a bilingual fetal environment during the last trimester of gestation and analyzed their capacity to encode voice pitch and vocalic formant structure information.

## Methods

### Participants

A sample of 131 newborns (mean age after birth = 38.32 ± 23.8 h) was recruited from *SJD Barcelona Children’s Hospital* in Barcelona (Spain) and divided into two groups based on a short retrospective questionnaire delivered to the babies’ mothers. Mothers were asked if they communicated using more than one language during the last 3 months of pregnancy and were instructed to report which languages they communicated in, provided they accounted for a minimum of 20% language usage time. Based on the collected responses, a total of 53 newborns were assigned to the group exposed to a monolingual fetal acoustic environment (MON; 27 females; mean gestational age = 39.93 ± 1.03 weeks; mean birth weight = 3,321 ± 272 g). A total of 76 newborns were assigned to the bilingual-exposed group (BIL; 33 females; mean gestational age = 39.71 ± 0.99 weeks; mean birth weight = 3,328 ± 327 g) after excluding two newborns, as their mothers were multilingual in Spanish, Catalan and English, being the third language used ≧20% of the time. Regarding the languages spoken by the bilingual mothers, all except one were Spanish—Other language and most of them were Spanish-Catalan bilinguals (77.3%). The other languages spoken were Arabic (6/75), English (1/75), Galician (1/75), German (1/75), Italian (2/75), Portuguese (2/75), Guaraní (2/75) and Romanian (2/75). On the other hand, newborns in the monolingual group were either exposed to Spanish (90.6%) or Catalan (9.4%).

No significant differences were found across groups in gestational age (U_(127)_ = 1868.500, *p* = 0.370), birth weight (t_(127)_ = −0.116, *p* = 0.908) and sex (χ^2^ = 0.710, *p* = 0.399). Maternal education level and musical exposure were assessed using a sociodemographic questionnaire (an English version of the sociodemographic questionnaire can be found in the Supplementary material). Groups did not differ in maternal educational level (χ^2^ = 1.992, *p* = 0.574), a key confounding factor associated with language acquisition and development (Hoff, 2003; Rowe, 2008) closely tied to the linguistic environment a fetus is exposed to. We also ascertained that groups did not differ in prenatal musical exposure [χ^2^ = 0.025, *p* = 0.874; see Arenillas-Alcón et al. (2023) for details], as it exerts a significant impact on speech encoding capacities at birth (Partanen et al., 2013b, 2022; Arenillas-Alcón et al., 2023).

All neonates obtained Apgar scores higher than 8 at 1 and 5 min of life and passed adequately the universal newborn hearing screening (UNHS) before the recruitment. According to the recommendations of the Joint Committee on Infant Hearing (2019), newborns born from high-risk gestations, after obstetric pathologies or any other kind of risk factors related to hearing impairment were excluded from the recruitment.

Additionally, as performed in previous research from our laboratory (Ribas-Prats et al., 2019, 2021, 2023; Arenillas-Alcón et al., 2021, 2023), both groups of newborns received a standard click-evoked auditory brainstem response (ABR) test to ensure the integrity of the auditory pathway. A click-stimulus, with a duration of 100 μs, was employed during the test, presented at a rate of 19.30 Hz with an intensity of 60 dB sound pressure level (SPL) until a total of 4000 artifact-free repetitions were collected. A prerequisite for participation in the experiment for all newborns was the successful identification of the wave V peak. This study was approved by the Ethical Committee of Clinical Research of the Sant Joan de Déu Foundation (Approval ID: PIC-53-17), and required the mothers to fill out a sociodemographic questionnaire and to sign an informed consent prior to the participation, in line with the Code of Ethics of the World Medical Association (Declaration of Helsinki).

### Stimulus

**Figure 1:**
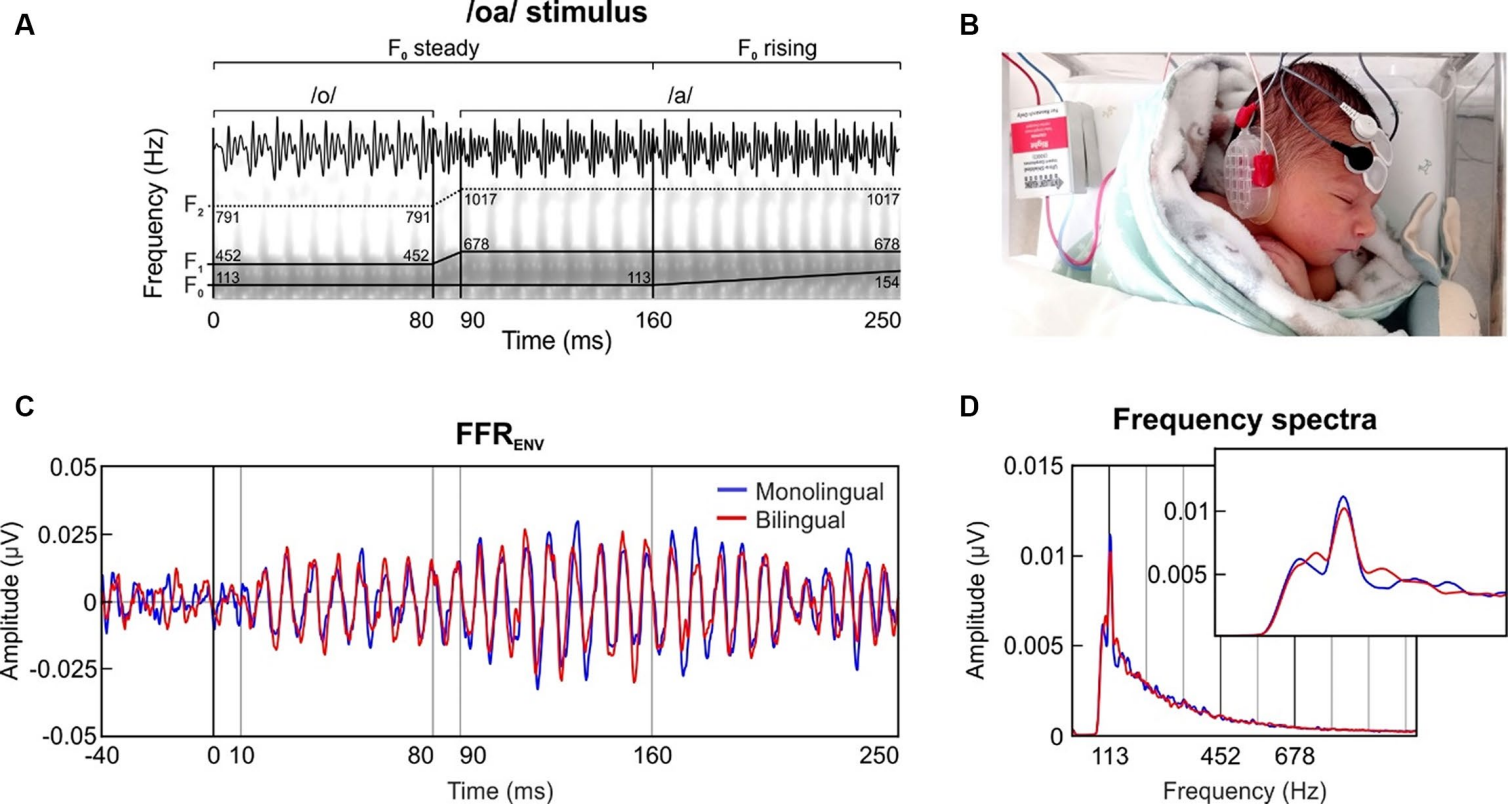
**(A)** Temporal and spectral representation of the two-vowel auditory stimulus /oa/, with traces indicating its fundamental frequency (F_0_) and formant structure (F_1_, F_2_). **(B)** Recording setup of the three disposable electrodes placed in a vertical montage (active located at Fpz, ground at forehead, references at the right mastoid). Baby’s photograph reproduced with the written consent of the neonate’s parents. **(C)** Grand-averaged waveform of the FFR_ENV_ in the time domain, retrieved separately for the group exposed to monolingual (blue) and bilingual (red) fetal acoustic environment. **(D)** Frequency spectra of the FFR_ENV_ extracted from the steady pitch section of the stimulus (10–160 ms). The inset zooms in a narrower frequency band to illustrate the effect around the F_0_ peak.

Neonatal FFRs were collected to a two-vowel stimulus with a rising pitch ending (/oa/; Arenillas-Alcón et al., 2021). The /oa/ stimulus was created in *Praat* (Boersma and Weenink, 2020) and had a total length of 250 ms divided into three different sections, according its fundamental frequency (F_0_) and its formant content (/o/ vowel section: 0–80 ms, F_0_ = 113 Hz, F_1_ = 452 Hz, F_2_ = 791 Hz; /oa/ formant transition section = 80–90 ms; /a/ vowel steady section = 90–160 ms, F_0_ = 113 Hz, F_1_ = 678 Hz, F_2_ = 1,017 Hz; /a/ vowel rising section = 160–250 ms, F_0_ = 113–154 Hz, F_1_ = 678 Hz, F_2_ = 1,017 Hz; Figure 1A).

The stimulus was designed with optimal parameters to study the frequency-following response, specially taking into account that due to the low-pass filter characteristics of the womb, fetuses are isolated from the mid and high frequency acoustic content of external sounds that characterizes most of the temporal fine structure of speech. The /oa/ stimulus used includes a pitch variation and two vowel sections with different formant structure based on relatively lower frequency harmonic components and suitable durations for accurate spectral analyses, which enable a proper assessment of speech sound temporal envelope and temporal fine structure encoding (Krizman and Kraus, 2019; Arenillas-Alcón et al., 2021). The relatively low F_0_ frequency, typical of a male speaker, was chosen to ensure a reliable measure of the neural representation of sound pitch (Krizman and Kraus, 2019) and the phonetic contrasts (/o/; /a/) belong to the phonetic repertoire of both Spanish and Catalan languages.

The /oa/ stimulus was presented at a rate of 3.39 Hz in alternating polarities and delivered monaurally to the right ear at 60 dB SPL of intensity with an earphone connected to a Flexicoupler disposable adaptor (Natus Medical Incorporated, San Carlos, CA).

### Procedure and data acquisition

After the successful completion of the UNHS, neonates were tested at the hospital room while they were sleeping in their bassinet. Three disposable Ag/AgCl electrodes were placed in a vertical montage configuration (active at Fpz, ground at forehead, reference at the right mastoid, ipsilateral to the auditory stimulation; as shown in Figure 1B), ensuring impedances below 7 kΩ. The presentation of click and speech stimuli was done by using a *SmartEP* platform connected to a *Duet* amplifier, which incorporated the *cABR* and the *Advanced Hearing Research* modules (Intelligent Hearing Systems, Miami, FL, United States).

The experimental procedure involved the recording of two blocks of click stimuli, followed by four blocks of 1000 artifact-free responses to the /oa/ stimulus. Any electrical activity surpassing ±30 μV threshold was automatically rejected until a total of 4,000 presentations was collected. The total mean duration of the recording session was approximately 25 min [2 click blocks × 2,000 repetitions × 51.81 ms SOA + 4 /oa/ blocks × 1,000 repetitions × 295 ms of stimulus-onset asynchrony (SOA)] including the duration of rejected sweeps. The continuous EEG signal was acquired at a sampling rate of 13,333 Hz with an online bandpass filter with cutoff frequencies from 30 to 1,500 Hz and online epoched from −40.95 ms (pre-stimulus period) to 249.975 ms.

### Data processing and analysis

Data epochs were bandpass filtered offline from 80 to 1,500 Hz and averaged separately per stimulus polarity. To highlight the encoding of the stimulus fundamental frequency (F_0_) and to reduce the contribution of cochlear microphonics, neural responses to the two opposite stimulus polarities were added [(Condensation + Rarefaction)/2], obtaining the envelope-following response (FFR_ENV_). Further, to emphasize the FFR components associated with the encoding of the stimulus temporal fine structure, such as the first formant (F_1_), while reducing the impact of envelope-related activity, the neural responses to alternating polarities were subtracted [(Condensation − Rarefaction)/2], yielding the temporal fine structure-following response (FFR_TFS_; Aiken and Picton, 2008; Krizman and Kraus, 2019). Considering the stimulus formant content, we focused our analyses exclusively on the spectral peaks that corresponded to F_1_ frequencies, as F_2_ frequencies fall at the limits of the spectral resolution of the FFR, resulting in elicited neural responses relatively weak and challenging to be accurately observed in newborns (Gorina-Careta et al., 2022). Detailed information regarding the analyzed parameters from the neonatal FFR can be found below. All parameters were computed using custom scripts in Matlab R2019b (The Mathworks Inc., 2019), developed in our laboratory and previously employed in similar analyses in former studies (Arenillas-Alcón et al., 2021).

#### Neural lag

Neural lag served as an indicator of the neural transmission delay within the auditory system, and was assessed to estimate the time passed from cochlear stimulus reception to the onset of neural phase-locking (Jeng et al., 2010; Liu et al., 2015; Ribas-Prats et al., 2019, 2021, 2023; Arenillas-Alcón et al., 2021, 2023). To calculate the neural lag, a cross-correlation analysis was computed between the auditory stimulus and the neural response. The neural lag was determined by identifying the time lag corresponding to the highest cross-correlation value within a time window of 3–13 ms.

#### Pre-stimulus root mean square (RMS) amplitude

The RMS of the pre-stimulus period was employed as a measure of the general magnitude of neural activity over time, and to dismiss electrophysiological disparities in the pre-stimulus region (Liu et al., 2015; White-Schwoch et al., 2015; Ribas-Prats et al., 2019, 2021, 2023; Arenillas-Alcón et al., 2023). This measure was computed by squaring each data point within the pre-stimulus region of the neural response (from −40 to 0 ms), calculating the mean of the squared values and subsequently obtaining the square root of the resulting average.

### Voice pitch encoding from FFR_ENV_

#### Spectral amplitude at F_0_

Spectral amplitude at F_0_ (113 Hz) was used as a quantitative measure of the neural phase-locking strength at the specific frequency of interest (White-Schwoch et al., 2015; Ribas-Prats et al., 2019, 2021, 2023; Arenillas-Alcón et al., 2021, 2023). It was computed by applying a fast Fourier transform (FFT; Cooley and Tukey, 1965) to obtain the frequency spectrum of the neural response during the steady pitch section of the stimulus (10–160 ms), and then calculating the average amplitude within a ± 5 Hz window centered around the peak of the stimulus F_0_.

#### Signal-to-noise ratio at F_0_

Signal-to-noise ratio (SNR) at F_0_ was analyzed to obtain an estimation of the relative spectral magnitude of the response, taking into account not only to the amplitude value at the F_0_ frequency peak (113 Hz) but also the noise levels at the surrounding frequencies. Therefore, the SNR was calculated by dividing the mean spectral amplitude within a ± 5 Hz frequency window centered at the peak of the frequency of interest (113 Hz) by the averaged mean amplitude within two additional 28 Hz wide frequency windows (flanks), centered at ±19 Hz from the frequency of interest (80–108 Hz and 118–146 Hz).

### Formant structure encoding from FFR_TFS_

#### Spectral amplitudes at F_1_ peaks

To assess spectral amplitudes at the specific spectral peaks regarding the stimulus F_1_ frequencies (452 Hz [/o/] and 678 Hz [/a/]), the neural responses corresponding to the /o/ section (10–80 ms time window) and the /a/ steady section (90–160 ms time window) were individually analyzed and the respective amplitudes within a ± 5 Hz window centered at the peak frequencies corresponding to the vowel formant centers were extracted. The transition from /o/ vowel to /a/ vowel was not analyzed due to its short duration (10 ms).

#### Signal-to-noise ratio at F_1_

To compute the relative spectral magnitude of the response at the stimulus F_1_ frequencies considering noise levels, SNRs at spectral peaks that correspond to the stimulus F_1_ frequencies (452 Hz and 678 Hz) were calculated separately on the /o/ and the /a/−steady sections. To do so, the SNR was calculated by dividing the mean spectral amplitude within a ± 5 Hz frequency window centered at the peak of the frequency of interest (452 or 678 Hz) by the averaged mean amplitude within two additional 28 Hz wide frequency windows (flanks), centered at ±26 Hz from the frequency of interest (for 452 Hz peak: 402–430 Hz and 474–502 Hz; for 678 Hz peak: 628–656 Hz and 700–728 Hz).

### Statistical analysis

Statistical analyses were conducted using Jamovi 2.3.26 (The Jamovi Project, 2023). Descriptive statistics were calculated, including the mean, standard deviation (SD), median, first quartile (Q_1_), third quartile (Q_3_), interquartile range (IQR), and minimum and maximum values, for each computed parameter within the two groups of newborns (MON; BIL).

To analyze the effects of prenatal bilingual exposure on neural transmission delay, pre-stimulus root mean square amplitude and voice pitch encoding depending on the normality of the data, two-tailed independent samples *t*-tests or Mann–Whitney U tests were conducted to evaluate significant differences between groups, with Cohen’s *d* being reported as the effect size. Kolmogorov–Smirnov test was used to assess the normal distribution of the samples.

The effects of prenatal bilingual exposure on formant structure encoding were analyzed with two repeated–measures ANOVAs with the factor Stimulus Section (/o/ section; /a/ section) as within-subjects factor and the factor Group (Monolingual; Bilingual) as between-subjects factor for each of the two formant amplitudes (452 and 678 Hz) separately. The Greenhouse–Geisser correction was applied when the assumption of sphericity was violated. Additional two-tailed independent samples Mann–Whitney U post-hoc tests were performed to examine the direction of the effects. Results were considered statistically significant when *p* < 0.05.

## Results

Frequency following responses (FFR) elicited by a two-vowel speech stimulus /oa/ (Figure 1A) were collected from a total sample of 129 newborns divided into two groups according to their prenatal fetal exposure to monolingual (MON) or bilingual (BIL) maternal speech. To comprehensively evaluate the neonates’ ability to encode the pitch and vowel formant structure of speech sounds, the neural responses to the fundamental frequency (F_0_) and the vowels’ first formant (F_1_) were analyzed considering the distinct sound characteristics of the different stimulus sections. All detailed descriptive statistics from the parameters analyzed can be found in Supplementary Table S1.

### Neural transmission delay

No significant differences were found across groups in neural lag (U_(127)_ = 1950.500, *p* = 0.763, Rank-biserial correlation = 0.032).

### Pre-stimulus root mean square (RMS) amplitude

There were no statistically significant differences observed between the groups with regards to the background neural activity preceding the auditory stimulation (U_(127)_ = 1914.000, *p* = 0.634, Rank-biserial correlation = 0.050).

### Voice pitch encoding (FFR_ENV_)

The grand-averaged FFR_ENV_ waveform for each group is illustrated in Figure 1C. To assess the robustness of the voice pitch representation, we analyzed the steady section (10–160 ms) of the /oa/ stimulus with a steady fundamental frequency (F_0_) of 113 Hz.

The grand-averaged spectral representation of the neonatal FFR extracted from each group is depicted in Figure 1D. No differences were found across groups in spectral amplitude at F_0_ computed using the steady pitch section of the stimulus (U_(127)_ = 1736.000, *p* = 0.184, Rank-biserial correlation = 0.138).

Yet, the statistical analyses performed on the F_0_ SNR, which represents the F_0_ relative spectral amplitude in relation with the spectral amplitude of the neighboring frequencies, revealed significant differences between groups, indicating that newborns exposed to a monolingual prenatal fetal environment exhibited significantly larger SNR values as compared to the bilingual exposed neonates (U_(127)_ = 1508.000, *p* = 0.016, Rank-biserial correlation = 0.251).

### Formant structure encoding (FFR_TFS_)

**Figure 2:**
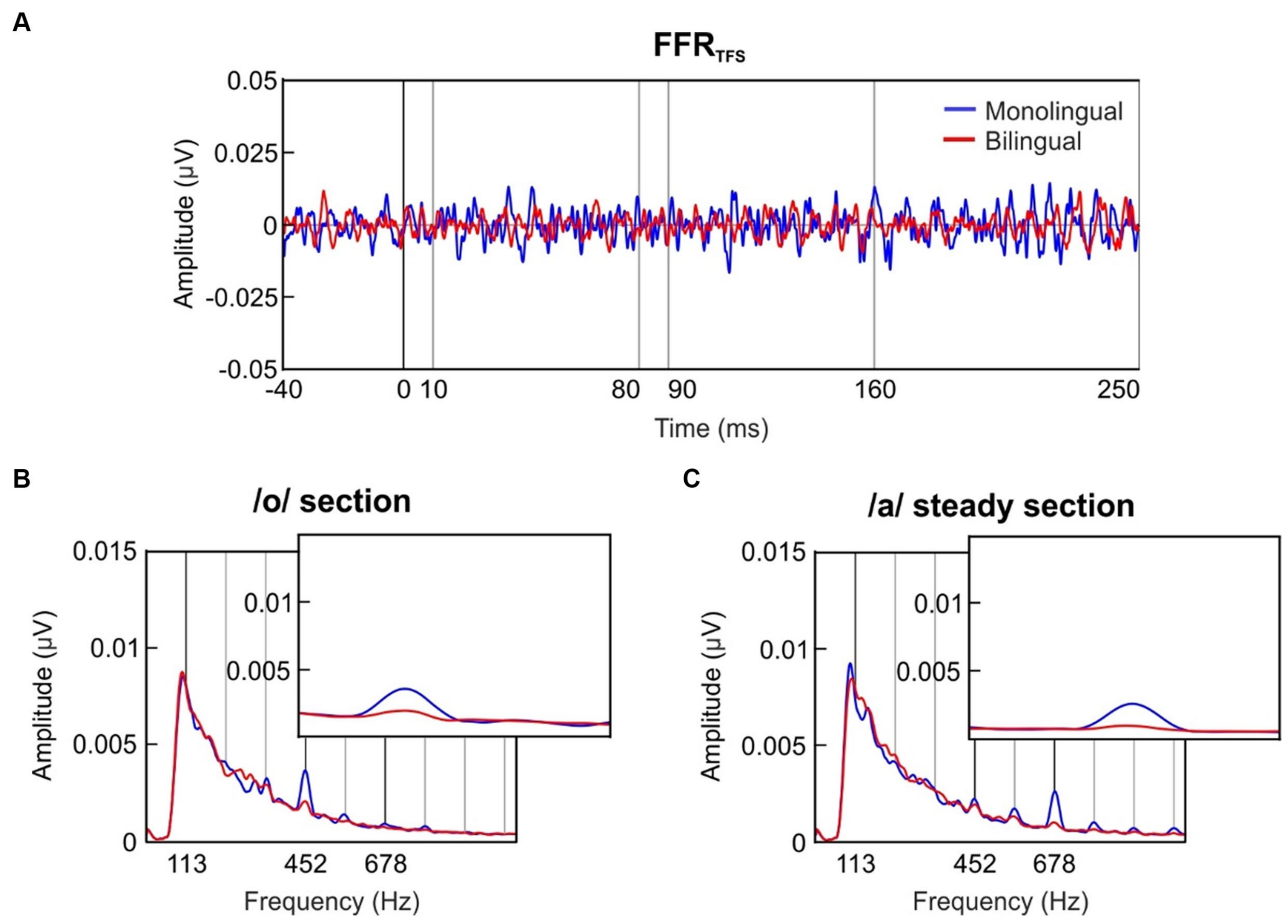
Formant structure encoding. **(A)** Grand-averaged waveform of the FFR_TFS_ in the time domain, retrieved separately for the group exposed to a monolingual fetal acoustic environment (blue) and the bilingual-exposed group (red). **(B)** Frequency spectra of the FFR_TFS_ extracted from the /o/ section of the stimulus (10–80 ms). The inset zooms in a narrower frequency band to illustrate the effect around the /o/ F_1_ peak (452 Hz) during the /o/ section. **(C)** Frequency spectra of the FFR_TFS_ extracted from the /a/ steady section of the stimulus (90–160 ms). The inset zooms in a narrower frequency band to illustrate the effect around the /a/ F_1_ peak (678 Hz) during the /a/ steady section.

The grand-averaged FFR_TFS_ waveform for each group is shown in Figure 2A. To evaluate the newborns’ ability to encode the formant structure of speech sounds, the /oa/ stimulus included two sections with the same voice pitch but different fine-structure. Specifically, the /o/ section (10–80 ms) was characterized by a center formant frequency (F_1_) of 452 Hz, and the /a/ steady section (90–160 ms) by a F_1_ frequency of 678 Hz. Spectral amplitudes were retrieved from the FFR_TFS_ separately from neural responses during the /o/ section and the /a/ steady-pitch section, selecting the spectral peaks corresponding to stimulus F_1_ frequencies.

**Figure 3:**
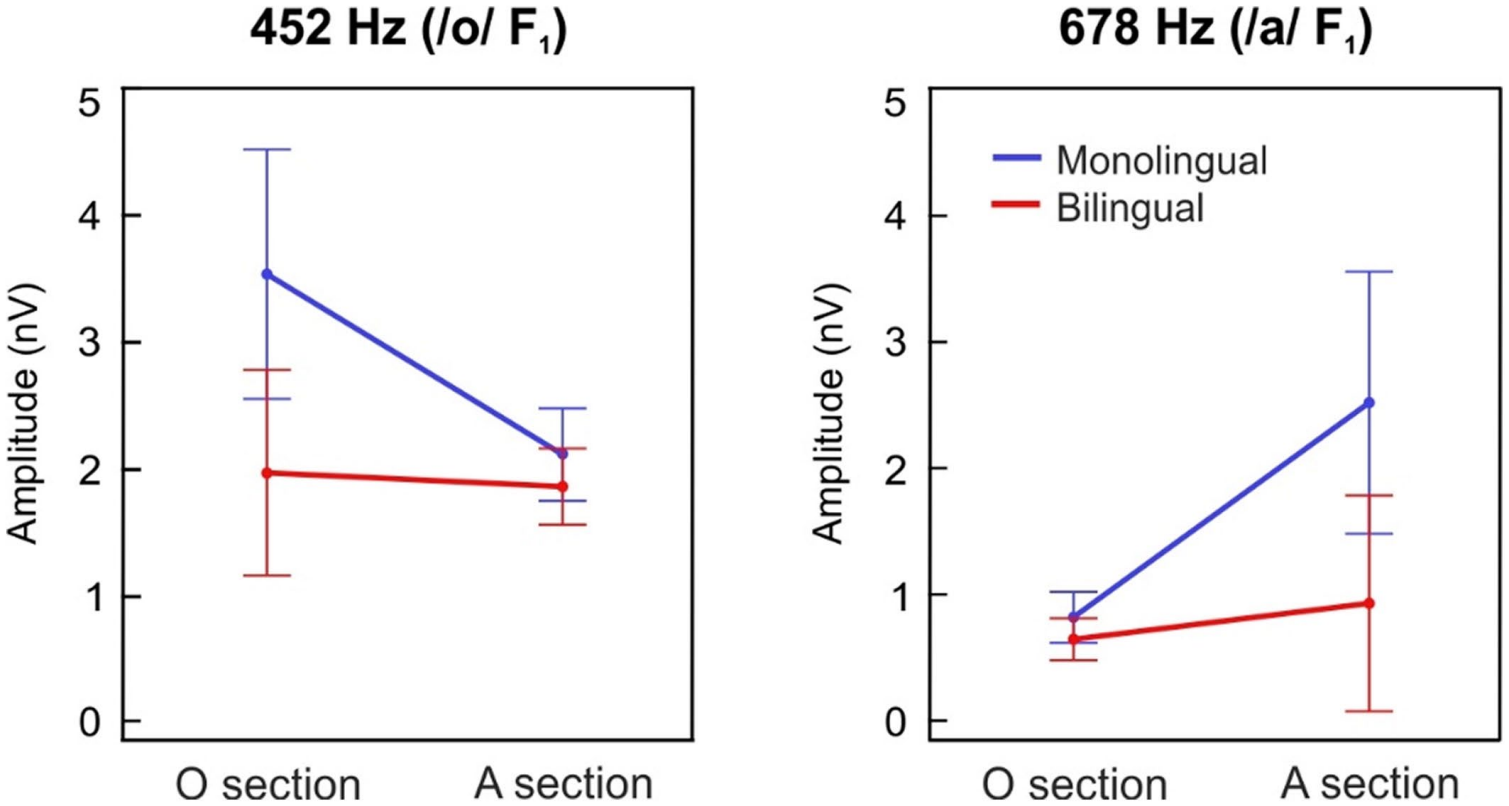
Spectral amplitudes at the first formant (F_1_). F_1_ spectral amplitudes at 452 Hz (left) and 678 Hz (right) during the /o/ section (10–80 ms) and the /a/ steady section (90–160 ms), plotted in blue and red lines for the monolingual and the bilingual-exposed newborns, respectively. Error bars represent 95% confidence intervals.

The grand-averages of the FFR_TFS_ spectral amplitudes during the /o/ section are illustrated in Figure 2B for each group separately, while the spectral representations during the /a/ steady section are depicted in Figure 2C. F_1_ spectral amplitudes during the /o/ section and the /a/ steady section are depicted in Figure 3 for each group at each formant center frequency (452 Hz, 678 Hz) separately.

When analyzing the effects of a prenatal maternal bilingual language exposure in formant spectral amplitude at 452 Hz (Figure 3, left panel), which corresponds to the F_1_ center frequency of the /o/ vowel, a main effect of group revealed significantly greater spectral amplitudes in the MON group as compared to the BIL (group main effect; *F*_(1,127)_ = 4.939, *p* = 0.028, ηp2 = 0.037). Moreover, a significantly larger spectral amplitude was observed during the /o/ section vs. /a/ steady section (stimulus section main effect; F_(1,127)_ = 7.580, *p* = 0.007, ηp2 = 0.056), thus indicating a proper encoding of the vowel /o/ in its corresponding stimulus section. Interestingly, a significant interaction of group per stimulus section was identified as well (interaction; F_(1,127)_ = 5.809, *p* = 0.017, ηp2 = 0.044), demonstrating that MON neonates showed significantly larger spectral amplitudes during the /o/ section at its corresponding formant frequency than BIL.

Similar results were observed when analyzing the effects of a prenatal maternal bilingual language exposure in the formant encoding at 678 Hz (Figure 3, right panel), which corresponds to the F_1_ center frequency of the /a/ vowel. A main effect of group revealed significantly greater spectral amplitudes in the MON group as compared to the BIL (group main effect; F_(1,127)_ = 5.01, *p* = 0.027, ηp2 = 0.038). Moreover, a significantly larger spectral amplitude at 678 Hz during the /a/ steady section vs. /o/ section was observed (stimulus section main effect; F_(1,127)_ = 10.93, *p* = 0.001, ηp2 = 0.079), thus indicating a proper encoding of the /a/ vowel in its corresponding stimulus section. Interestingly, a significant interaction of group per stimulus section was also identified (interaction; F_(1,127)_ = 5.812, *p* = 0.017, ηp2 = 0.044), demonstrating that the MON group exhibited higher spectral amplitudes during the /a/ steady section at its corresponding frequency than the BIL.

The same pattern of results was obtained when comparing the relative spectral amplitude of the response at the stimulus F_1_ frequencies taking into account the neural response to the neighboring frequencies. When analyzing the effects of a fetal maternal bilingual language exposure in SNR at 452 Hz, which corresponds to the F_1_ of the /o/ vowel, a main effect of group revealed significantly greater spectral amplitudes in the MON group as compared to the BIL (group main effect; F_(1,127)_ = 8.301, *p* = 0.005, ηp2 = 0.061). Moreover, a significantly larger spectral amplitude was observed during the /o/ section vs. /a/ steady section (stimulus section main effect; F_(1,127)_ = 7.517, *p* = 0.007, ηp2 = 0.056). A significant interaction of group per stimulus section was identified as well (interaction; F_(1,127)_ = 7.304, *p* = 0.008, ηp2 = 0.054).

Similar effects were observed when analyzing the effects of a prenatal bilingual environment in the formant SNR at 678 Hz, which corresponds to the frequency of the /a/ vowel. A main effect of group revealed significantly greater spectral amplitudes in the MON group as compared to the BIL (group main effect; F_(1,127)_ = 7.127, *p* = 0.009, ηp2 = 0.053). Moreover, a significantly larger spectral amplitude at 678 Hz during the /a/ steady section vs. /o/ section was observed (stimulus section main effect; F_(1,127)_ = 22.072, *p* < 0.001, ηp2 = 0.148). Finally, a significant interaction of group per stimulus section was also identified (interaction; F_(1,127)_ = 10.330, *p* = 0.002, ηp2 = 0.075).

## Discussion

The present study investigated the impact of maternal bilingual speech during pregnancy on the neural encoding of speech pitch and vowel formant structure in neonates. A total sample of 129 healthy-term newborns was divided into two groups according to their monolingual or bilingual prenatal exposure during the last trimester of gestation, as reported by their mothers through a questionnaire. FFRs elicited to a two-vowel speech stimulus /oa/ (Arenillas-Alcón et al., 2021) were recorded to assess the neural responses to the stimulus’ fundamental frequency (F_0_ = 113 Hz; related to voice pitch encoding) and the first formant of each vowel (/o/ F_1_ = 452 Hz; /a/ F_1_ = 678 Hz; related to vowel formant structure encoding). Our results revealed that the neural representation of pitch, as indexed by the spectral amplitude of the FFR_ENV_ at the stimulus F_0_, did not differ between monolingual and bilingual exposure groups, but monolingually exposed neonates exhibited a higher signal-to-noise ratio (SNR) at the F_0_ spectral peak, suggesting the contribution of a higher spectral noise at neighboring frequencies in the bilingual group. Additionally, monolingually exposed neonates exhibited larger spectral amplitudes and SNRs of the FFR_TFS_ at the formant peak frequencies (F_1_) of the speech stimulus used, indicating a stronger encoding of vocalic structure. Furthermore, no significant group differences were observed in neural lag and pre-stimulus root mean square (RMS) amplitude, implying comparable neural transmission delays and absence of a distinct overall neural activity prior to the auditory stimulation. Together, these findings provide novel insights into the effects of prenatal language exposure on the neural encoding of speech sounds at birth.

Pitch is a crucial attribute in the perception of periodic speech sounds, as it conveys prosodic information, facilitates speaker recognition and speech segmentation, accelerates phoneme acquisition in tonal languages, helps with language comprehension in noisy environments and even contributes to the perception of the emotional state in a conversation (Musacchia et al., 2007; Benavides-Varela et al., 2012; Partanen et al., 2013a; Plack et al., 2014; Gervain, 2018; Cabrera and Gervain, 2020; Arenillas-Alcón et al., 2021; Ribas-Prats et al., 2021). The fact that neural mechanisms underlying voice pitch encoding are already mature at birth (Jeng et al., 2011; Ribas-Prats et al., 2019; Cabrera and Gervain, 2020; Arenillas-Alcón et al., 2021) suggests that pitch may play a crucial role in the very first stages of language acquisition (Jeng et al., 2016). Going a step further, pitch could provide a neural synchrony channel onto which separate neural representations of other speech features would anchor as parts of an ensemble that would, ultimately, give rise to a coherent percept (Eggermont, 2001).

Previous studies demonstrated that pitch and pitch contour discrimination drastically improve with training (e.g., Carcagno and Plack, 2017). In this regard, growing up in a bilingual environment, which is characterized as more demanding, dynamic, phonologically rich and requiring heightened attention to all linguistic input, is related to a strengthened neural representation of pitch (Krizman et al., 2012, 2015). Different languages have distinct overall height pitch levels. For example, Catalan was observed to have a higher pitch compared to Spanish (Marquina Zazura, 2011); Polish was found to have a higher pitch compared to American English (Majewski et al., 1972); Mandarin, a higher pitch than English (Keating and Kuo, 2012); Japanese, a higher pitch than Dutch (Van Bezooijen, 1995); or Slavic languages, a higher pitch than Germanic ones (Andreeva et al., 2014). Further, speakers of two phonologically similar dialects exhibit differences in their height pitch levels (e.g., two different dialects of Mandarin; Deutsch et al., 2009).

Yet, pitch height is not the only element that contributes significantly to the distinctiveness of a particular language. The intonational patterns, which are the rising and falling patterns of pitch that convey meaning and contribute to the rhythm of speech, may differ between the different languages. When a speaker switches between languages they naturally adjust the specific contours, pitch ranges, and other prosodic features to conform to the norms of the target language, and many linguistic features such as intonation, may affect the mean fundamental frequency of speech (Järvinen et al., 2013). This adjustment helps maintaining communicative clarity and aligns with the phonetic characteristics of the language being spoken (Mary and Yegnanarayana, 2008; Passoni et al., 2022).

With continued exposure to these complex linguistic contexts, the auditory system gradually becomes finely tuned to process sound more efficiently (Krizman et al., 2012). Thus, individuals with years of exposure and interaction with bilingual environments develop enhanced flexibility and speech-encoding abilities. Most notably, previous studies have shown that bilingual individuals, particularly females, exhibit different pitch frequency ranges depending on the language they speak (Ordin and Mennen, 2017). As both pitch and the intonational patterns of the languages are different, and the prosodic elements of speech which include pitch contours, rhythm, and stress (Moon and Fifer, 2000) are acoustic features reliably transmitted through the womb (Gerhardt and Abrams, 2000; May et al., 2011), bilingual mothers provide their children with a higher pitch variability *in utero*.

Considering the reviewed literature, if the developing auditory system of a fetus, who underwent approximately 3 months of noninteractional exposure to degraded speech, responded to acoustic exposure as the mature one, we would expect newborns from bilingual mothers to exhibit a higher neural encoding of voice pitch. But our results showed otherwise. We found no differences across groups in FFR_ENV_ spectral amplitudes at F_0_, which aligns with the idea that pitch processing mechanisms are already mature at birth. Yet, we observed a decreased SNR at the F_0_ in newborns who were prenatally exposed to a bilingual environment. We attempt to reconcile our seemingly contradicting results by hypothesizing that the higher spectral amplitudes found in bilingually exposed neonates at F_0_ neighboring frequencies reflect an increased sensitivity to a wider range of pitch frequencies without yet generating a particularly strong response at any of them.

This view aligns with research on perceptual phonetic development, especially when growing in bilingual environments. Previous studies demonstrated that experience with language shapes infants’ abilities to process speech sounds and, with age, the newborn’s ability to differentiate phonetic distinctions becomes more language-specific (Kuhl et al., 2006; Saffran et al., 2006; Gervain and Werker, 2008; Bosch and Sebastián-Gallés, 2010). At birth all infants possess the ability to perceive all sound distinctions used in languages as they are sensitive to the basic rhythmic differences between languages (Nazzi et al., 1998; Byers-Heinlein et al., 2010). Around 3–4 months of age infants are sensitive to rhythmic differences between languages that go beyond their belonging to the three basic rhythmic classes (Bosch and Sebastián-Gallés, 2010; Molnar et al., 2014) and by the age of 6 months monolingual infants’ ability to perceive speech becomes tailored to their native language. Infants exposed to two languages are also able to discriminate the sound contrasts of both their languages, but this occurs only at the end of their first year (Bosch and Sebastián-Gallés, 2003; Sundara et al., 2008; for review see Hammer et al., 2014).

Yet, the early prenatal impact of language goes beyond language discrimination. As reviewed in the introduction, newborns prefer their mother’s voice over other female voices (DeCasper and Fifer, 1980), their communicative cries reflect the prosody of the language they heard *in utero* (Mampe et al., 2009) and can recognize stories heard during pregnancy (DeCasper and Spence, 1986). Moreover, previous studies also demonstrated that differences in prenatal language exposure modulate perceptual grouping biases at birth (Abboub et al., 2016) and suggest that hearing pitch contrasts before birth may influence pitch-based grouping preferences and may lead to a stable bias at birth. Thus, despite the discrimination (or no discrimination) of languages at birth, prenatal language exposure modulates the processing of speech sounds. Our findings align with the suggested hypothesis that being bilingual confers a greater perceptual flexibility (Abboub et al., 2016), as we observed in bilingually exposed newborns an increased sensitivity to a wider range of pitch frequencies.

Our results also reveal a modulation of the neural encoding of vowel formants (F_1_) depending on prenatal linguistic exposure. In particular, monolingual-exposed neonates exhibited higher spectral amplitudes at the corresponding formant frequencies of the stimulus’ /o/ and steady−/a/ vowels. In a previous study, we found that while the neural encoding of pitch was adult-like at birth, formant encoding was still immature (Arenillas-Alcón et al., 2021). As vowel formant center frequencies are language specific and stable regardless of voice pitch variation, which also presents slight modulations in monolingual individuals during natural speaking, the auditory system of a monolingual-exposed fetus receives a more consistent phonetic repertoire than that of a bilingual-exposed. This would possibly lead to a more effective and accurate encoding of the specific language vowel sound characteristics at birth. Simply put, monolingual newborns seem to have an advantage in processing the specific sounds of their mother tongue, a finding previously attributed to postnatal linguistic exposure (Kuhl, 2010). Our findings thus highlight the greater variability of acoustic speech inputs to which the fetus of bilingual mothers would be exposed and therefore suggest the need for bilinguals to develop a different phonological representation for each of the languages (Sebastian-Gallés et al., 2006). Further investigation into the developmental trajectories of auditory processing in different populations of newborns, with different prenatal auditory experiences, and using language-specific phonetic contrasts (e.g., Catalan contrasts such as /e - ɛ/), which are especially difficult –when not impossible– to detect for Spanish-monolinguals (Pallier et al., 1997, 2001), may shed more light on this issue.

Despite being confident about our results due to the abovementioned reasons, we are fully aware of a number of limitations of our study: language exposure was assessed by a short (approx. 5 min answer time), retrospective questionnaire provided at the time of delivery, with a spoken description of the content of the questionnaire. This poses, at least, two factors not adequately controlled. First, the actual frequency in which mothers spoke any of the two languages, as we rely only on their reports referring to the last trimester of pregnancy. Furthermore, although a minimum period of usage time had to occur to be considered as valid, the questionnaire did not address the exact amount of language usage within a day. Future studies should address these limitations, for instance, by collecting large amounts of data from a maternal diary of language usage during the last trimester of pregnancy and include an additional language abilities test (such as LEAP-Q; Marian et al., 2007) to evaluate the putative link between F_0_ encoding abilities in newborns and maternal language usage percentage.

Overall, our findings emphasize the potential importance of prenatal linguistic exposure in shaping the neural mechanisms underlying language acquisition and highlight the sensitivity of the FFR in capturing these subtle changes. The results add to a growing body of research that suggests a role for prenatal fetal experiences in modeling language acquisition (Moon et al., 2012; Partanen et al., 2013b; Gervain, 2015, 2018; Arenillas-Alcón et al., 2023). Furthermore, they also highlight the importance of considering prenatal language exposure in developmental studies about language acquisition, a factor that is not routinely measured and reported, and that may contribute to divergent findings.

## Conclusion

The present study contributes significant insights into the impact of prenatal bilingual exposure on the neural encoding of speech sounds at birth, thereby increasing our knowledge of the early stages of language acquisition. The observed differences in the encoding of voice pitch and formant structure depending on prenatal linguistic exposure highlight the remarkable plasticity and learning potential of the human brain even before birth, emphasizing the complex interaction between genetic and environmental factors in shaping our cognitive abilities and linguistic development.

## Data availability statement

The data supporting the conclusions of this article will be made available upon request by the authors, without undue reservation.

## Ethics statement

The studies involving humans were approved by Ethical Committee of Clinical Research of the Sant Joan de Déu Foundation (Approval ID: PIC-53-17). The studies were conducted in accordance with the local legislation and institutional requirements. Written informed consent for participation in this study was provided by the participants’ legal guardians/next of kin.

## Author contributions

NG-C: Writing – review & editing, Writing – original draft, Software, Methodology, Investigation, Formal analysis, Data curation, Conceptualization. SA-A: Writing – review & editing, Writing – original draft, Visualization, Software, Methodology, Investigation, Formal analysis, Data curation, Conceptualization. MP: Investigation, Writing – review & editing, Methodology. AM-S: Writing – review & editing, Methodology, Investigation. SI-K: Writing – review & editing, Methodology, Investigation. JC-F: Writing – review & editing, Supervision, Methodology, Conceptualization. MG-R: Writing – review & editing, Resources, Funding acquisition. CE: Writing – review & editing, Supervision, Resources, Methodology, Funding acquisition, Conceptualization.
