# Supplementary material for: Exposure to bilingual or monolingual maternal speech during pregnancy affects the neurophysiological encoding of speech sounds in neonates differently
Source: Front Hum Neurosci. 2024 May 22;18:1379660. doi: 10.3389/fnhum.2024.1379660 (PMC11150635; doi:10.3389/fnhum.2024.1379660)
Supplement: Supplementary file 1 [file Data_Sheet_1.docx]

**Exposure to bilingual or monolingual maternal speech during pregnancy affects the neurophysiological encoding of speech sounds in neonates differently**

Natàlia Gorina-Careta^1,2,3,❖^, Sonia Arenillas-Alcón^1,2,3,❖^, Marta Puertollano^1,2,3^, Alejandro Mondéjar-Segovia^1,2^, Siham Ijjou-Kadiri^1,2^, Jordi Costa-Faidella^1,2,3,^*, María Dolores Gómez-Roig^3,4^ & Carles Escera^1,2,3,^*

,

^1^ Brainlab – Cognitive Neuroscience Research Group. Department of Clinical Psychology and Psychobiology, University of Barcelona (Catalonia, Spain)

^2^ Institute of Neurosciences, University of Barcelona (Catalonia, Spain)

^3^ Institut de Recerca Sant Joan de Déu, Santa Rosa 39-57, 08950 Esplugues de Llobregat (Catalonia, Spain)

^4^ BCNatal – Barcelona Center for Maternal Fetal and Neonatal Medicine (Hospital Sant Joan de Déu and Hospital Clínic), University of Barcelona (Catalonia, Spain)

^❖^ Both authors contributed equally to the preparation of the present work

*** Corresponding authors:**

*Carles Escera*

Brainlab - Cognitive Neuroscience Research Group

Department of Clinical Psychology and Psychobiology

University of Barcelona

P. Vall d'Hebron 171, 08035 Barcelona

Catalonia-Spain

cescera@ub.edu

*Jordi Costa-Faidella*

Brainlab - Cognitive Neuroscience Research Group

Department of Clinical Psychology and Psychobiology

University of Barcelona

P. Vall d'Hebron 171, 08035 Barcelona

Catalonia-Spain

jcostafaidella@ub.edu

**TABLES**

**Supplementary Table 1**

**Table 1. Descriptive statistics for MON** (n=53) **and BIL** (n=76) **groups in FFR parameters**: neural lag, Root-Mean-Square from pre-stimulus section, spectral amplitude at F_0_ and F_1_ peaks, Signal-to-noise ratio at F_0_ and F_1_ peaks.

| Measure | Mean | SD | Median | Q_1_ | Q_3_ | IQR | Minimum | Maximum |
| --- | --- | --- | --- | --- | --- | --- | --- | --- |
| **Neural lag** (ms) |  |  |  |  |  |  |  |  |
| Monolingual | 7.913 | 1.233 | 7.950 | 7.275 | 8.625 | 1.350 | 4.200 | 11.025 |
| Bilingual | 7.994 | 1.393 | 7.800 | 6.975 | 8.681 | 1.706 | 5.325 | 12.975 |
|  |  |  |  |  |  |  |  |  |
| **Pre-stimulus RMS** (nV) |  |  |  |  |  |  |  |  |
| Monolingual | 30.186 | 15.723 | 26.945 | 18.861 | 35.880 | 17.020 | 13.533 | 88.444 |
| Bilingual | 29.638 | 11.775 | 29.009 | 21.013 | 34.841 | 13.828 | 13.556 | 71.361 |
|  |  |  |  |  |  |  |  |  |
| **F_0_ Spectral Amplitude** (nV) |  |  |  |  |  |  |  |  |
| Monolingual | 10.015 | 5.184 | 9.041 | 6.556 | 12.270 | 5.713 | 1.503 | 26.110 |
| Bilingual | 9.108 | 4.932 | 7.652 | 6.063 | 11.139 | 5.076 | 2.568 | 23.960 |
| **SNR F_0_** |  |  |  |  |  |  |  |  |
| Monolingual | 2.063 | 0.834 | 1.960 | 1.435 | 2.674 | 1.240 | 0.627 | 4.185 |
| Bilingual | 1.735 | 0.753 | 1.736 | 1.217 | 2.072 | 0.855 | 0.522 | 3.831 |
|  |  |  |  |  |  |  |  |  |
| **F_1_ Spectral Amplitude /o/ section at 452 Hz** (nV) |  |  |  |  |  |  |  |  |
| Monolingual | 3.537 | 5.375 | 2.564 | 1.167 | 3.699 | 2.532 | 0.118 | 35.135 |
| Bilingual | 1.972 | 1.535 | 1.533 | 0.990 | 2.365 | 1.375 | 0.369 | 9.263 |
|  |  |  |  |  |  |  |  |  |
| **F_1_ Spectral Amplitude /a/ steady section at 452 Hz** (nV) |  |  |  |  |  |  |  |  |
| Monolingual | 2.118 | 1.373 | 1.863 | 1.358 | 2.512 | 1.153 | 0.279 | 7.869 |
| Bilingual | 1.877 | 1.328 | 1.731 | 0.946 | 2.434 | 1.489 | 0.242 | 9.405 |
|  |  |  |  |  |  |  |  |  |
| **F_1_ Spectral Amplitude /o/ section at 678 Hz** (nV) |  |  |  |  |  |  |  |  |
| Monolingual | 0.819 | 0.963 | 0.599 | 0.398 | 0.935 | 0.537 | 0.080 | 6.424 |
| Bilingual | 0.648 | 0.548 | 0.557 | 0.375 | 0.743 | 0.368 | 0.120 | 4.481 |
|  |  |  |  |  |  |  |  |  |
| **F_1_ Spectral Amplitude /a/ steady section at 678 Hz** (nV) |  |  |  |  |  |  |  |  |
| Monolingual | 2.519 | 5.948 | 1.063 | 0.599 | 1.907 | 1.309 | 0.167 | 38.348 |
| Bilingual | 0.915 | 0.730 | 0.750 | 0.360 | 1.347 | 0.987 | 0.061 | 5.068 |
|  |  |  |  |  |  |  |  |  |
| **SNR /o/ section at 452 Hz** |  |  |  |  |  |  |  |  |
| Monolingual | 2.749 | 3.562 | 1.462 | 0.837 | 3.247 | 2.410 | 0.085 | 21.957 |
| Bilingual | 1.442 | 0.879 | 1.240 | 0.760 | 1.932 | 1.172 | 0.285 | 4.854 |
|  |  |  |  |  |  |  |  |  |
| **SNR /a/ steady section at 452 Hz** |  |  |  |  |  |  |  |  |
| Monolingual | 1.738 | 1.460 | 1.272 | 0.830 | 2.031 | 1.201 | 0.254 | 6.946 |
| Bilingual | 1.434 | 0.931 | 1.297 | 0.731 | 1.927 | 1.196 | 0.154 | 5.207 |
|  |  |  |  |  |  |  |  |  |
| **SNR /o/ section at 678 Hz** |  |  |  |  |  |  |  |  |
| Monolingual | 1.331 | 1.481 | 0.932 | 0.625 | 1.723 | 1.099 | 0.161 | 10.369 |
| Bilingual | 1.115 | 0.669 | 0.928 | 0.729 | 1.278 | 0.549 | 0.179 | 3.167 |
|  |  |  |  |  |  |  |  |  |
| **SNR /a/ steady section at 678 Hz** |  |  |  |  |  |  |  |  |
| Monolingual | 3.492 | 5.694 | 1.671 | 0.918 | 3.261 | 2.344 | 0.267 | 32.254 |
| Bilingual | 1.520 | 1.086 | 1.349 | 0.726 | 1.862 | 1.136 | 0.149 | 6.513 |

SD = standard deviation. Q_1_ = first quartile (25th percentile). Q_3_ = third quartile (75th percentile). IQR = interquartile range.

**SUPPLEMENTARY MATERIAL - APENDIX**

**SOCIODEMOGRAPHIC, NOISE AND MUSICAL QUESTIONNAIRE (English translation).**

**A. SOCIODEMOGRAPHIC DATA**

1. Current Full Address: __
2. Place of birth: __
3. Ethnicity:

European / Maghrebi /African-American / Latino / Chinese / Asian-Philippine / Indian-Pakistani / Other, which one? __

1. Education:

  Primary school or less / Secondary school / Baccalaureate, vocational training or equivalent / University degree

1. Employment status:

  Unemployed / Self-employed / Salaried

1. Occupation: __
2. Net monthly family income:

< 1,000€ / 1,000€ - 2,000€ / 2,000€ - 3,000€ / > 3,000€

1. Maternal Language:

**B. SOCIODEMOGRAPHIC DATA OF THE PARTNER**

1. Place of birth: __
2. Ethnicity:

European / Maghrebi /African-American / Latino / Chinese / Asian-Philippine / Indian-Pakistani / Other, which one? __

1. Education:

  Primary school or less / Secondary school / Baccalaureate, vocational training or equivalent / University degree

1. Employment status:

  Unemployed / Self-employed / Salaried

1. Occupation: __

**C. QUESTIONNAIRE OF NOISE EXPOSURE IN PREGNANT WOMEN**

The following part of the questionnaire is intended to assess the amount of noise you have been exposed to in your work environment during the last trimester of your pregnancy. To do so, we ask you to answer the following questions. We remind you that any information provided will be kept confidential.

1. Did you regularly communicate in more than one language (take into account a minimum of 20% usage time)?

Yes / No

If yes, please answer the following questions. If no, continue with question 2.

- 1. Which languages did you use? __

1. During the last three months of pregnancy, were you working?

Yes / No

If yes, please answer the following questions. If no, continue with question 3.

- 1. How many hours per week? __

1. Indicate the duration of the medical leave, if any:

< 21 days / > 21 days / If you remember the exact number of days, please indicate: __

1. What is the title of the job you perform? __

**D. QUESTIONNAIRE OF MUSICAL EXPOSURE IN PREGNANT WOMEN**

The following part of the questionnaire aims to evaluate the amount of music your baby has been exposed to during the last trimester of pregnancy. To do so, we ask you to answer the following questions taking into account that all of them refer to musical exposure WITHOUT HEADPHONES. We remind you that any provided information will be treated with absolute confidentiality.

**Musical practice**

1. Did you play any musical instrument during the last trimester of pregnancy?

Yes / No

If yes, please answer the following questions. If no, continue with question 2.

- 1. Specify which instrument/s you played: __
  2. Indicate how frequently you played each one of the instruments:

Daily / Weekly / Once every two weeks / Monthly

1.3. What type of music did you usually play? In case you played several musical genres, please enumerate them from most to least practiced (being 1 the one you spent most time playing).

Classical / Pop/rock / Children's songs or lullabies / Other/s: __

1. Did you sing during the last 3 months of pregnancy?

Yes / No

If yes, please answer the following questions. If no, continue with question 2.

2.1. Indicate how frequently you sang:

Daily / Weekly / Once every two weeks / Monthly

2.2. What type of music did you usually sing? In case you sang several musical genres, please enumerate them from most to least practiced (being 1 the one you spent most time singing).

Classical / Pop/rock / Children's songs or lullabies / Other/s: __

**Musical exposure**

Please, remember we ask you to answer the following questions taking into account the last trimester of your pregnancy.

1. Did you listen to music with speakers (WITHOUT headphones) during the last 3 months?

Yes / No

3.1. Indicate how frequently you listened to music WITHOUT headphones.

Daily / Weekly / Once every two weeks / Monthly

3.2. What type of music did you usually listen to? In case you listened to several musical genres, please enumerate them from most to least listened (being 1 the one you spent most time listening to).

Classical / Pop/rock / Children's songs or lullabies / Other/s: __
